# Identification of Canine Pyometra-Associated Metabolites Using Untargeted Metabolomics

**DOI:** 10.3390/ijms232214161

**Published:** 2022-11-16

**Authors:** Hui-Hua Zheng, Chong-Tao Du, Yu-Zhu Zhang, Chao Yu, Rong-Lei Huang, Xin-Yue Tang, Guang-Hong Xie

**Affiliations:** College of Veterinary Medicine, Jilin University, Changchun 130062, China

**Keywords:** dog, canine pyometra, endometrium, metabolites, untargeted metabolomics

## Abstract

Canine pyometra frequently occurs in middle-aged to older intact bitches, which seriously affects the life of dogs and brings an economic loss to their owners. Hence, finding a key metabolite is very important for the diagnosis and development of a new safe and effective therapy for the disease. In this study, dogs with pyometra were identified by blood examinations, laboratory analyses and diagnostic imaging, and fifteen endometrium tissues of sick dogs with pyometra and fifteen controls were collected and their metabolites were identified utilizing a UHPLC-qTOF-MS-based untargeted metabolomics approach. The results indicated that the elevated inflammatory cells were observed in dogs with pyometra, suggesting that sick dogs suffered systemic inflammation. In the untargeted metabolic profile, 705 ion features in the positive polarity mode and 414 ion features in the negative polarity mode were obtained in endometrium tissues of sick dogs with pyometra, with a total of 275 differential metabolites (173 in positive and 102 in negative polarity modes). Moreover, the multivariate statistical analyses such as PCA and PLS-DA also showed that the metabolites were significantly different between the two groups. Then, these differential metabolites were subjected to pathway analysis using Metaboanalyst 4.0, and Galactose metabolism, cAMP signaling pathway and Glycerophospholipid metabolism were enriched, proving some insights into the metabolic changes during pyometra. Moreover, the receiver operating characteristic curves further confirmed kynurenic acid was expected to be a candidate biomarker of canine pyometra. In conclusion, this study provided a new idea for exploring early diagnosis methods and a safe and effective therapy for canine pyometra.

## 1. Introduction

The domestic dog is the most common companion animal for mankind and a model animal for biomedical research. Due to the increase in the dog population and the prolongation of a lifetime, the incidence of various diseases has been increasing in recent years, especially reproductive disorders. Among them, pyometra belongs to a common disease in countries where elective spaying is not routinely performed, and is characterized by an acute or chronic suppurative bacterial infection of the uterus post estrum with an accumulation of inflammatory exudate in the uterine lumen and a variety of clinical and pathologic manifestations, locally and systemically, even leading to the death of dogs [1]. Additionally, it has a morbidity of 19% in intact female dogs before 10 years of age, with a diagnosis rate of 20% in older bitches and more than 50% in certain high-risk breeds [2,3], which seriously threatens the life and health of dogs. At present, several methods are used to diagnose canine pyometra in clinical veterinary, including uterus symptom examinations, blood routine, blood biochemical examinations, C-reactive protein (CRP), ultrasonography exam and vaginal smears [1,4,5]. The histopathological examination is usually combined to further determine pathological changes in the canine uterus [6]. Generally, surgical ovariohysterectomy (OHE) is the most common treatment for canine pyometra [7]. Although this surgical treatment can decrease the morbidities of pyometra and canine mammary tumors, the risk of certain diseases may be increasing, such as cancers, orthopedic diseases, obesity and urinary incontinence [8,9,10,11,12]. Additionally, the operation risk of old dogs is high compared with that of adult dogs. Therefore, it is necessary to find specific biomarkers and develop a safe and efficient treatment or prevention of canine pyometra.

The biomarkers can provide a diagnosis basis and therapeutic window for specific diseases, such as canine mammary tumors, canine inflammatory enteropathies, chronic kidney disease in dogs and cardiovascular disease in humans [13,14,15,16]. To seek novel biomarkers and biochemical pathways for enhancing the early detection of disease, metabolomics is rapidly applied in the medicine area because it can conduct the quantitative analysis of circulating small molecule metabolites which may involve in the pathogenesis of the disease [16,17,18,19]. At present, metabolomics studies have successfully confirmed biomarkers for diagnosis, progression, and treatment for certain diseases, for example, cancer, diabetes and autoimmunity [20,21,22]. In addition, metabolites also have a major part to play in biological systems, and their difference analysis between unperturbed and perturbed pathways is conducive to identifying the physiological and pathological status of disease [23]. The study conducted by Ortiz et al. suggested the association of metabolites (amino acids, lipids, organic acids and other organic compounds) with endometriosis [24]. Similar to endometriosis, metabolites may also play an essential role in the early diagnosis, the improvement of clinical outcomes and the prevention of further progression of canine pyometra. However, the metabolites of the healthy canine uterus and ill canine uterus with pyometra are unclear so far.

Therefore, in the current study, we collected the uteruses of healthy dogs and ill dogs suffering from pyometra, with an identification of the clinical, blood and histopathological examination, and then compared the metabolome of healthy dogs and diseased dogs with pyometra utilizing a ultra-high performance liquid chromatography-quadrupole time offlight mass spectrometry (UHPLC-qTOF-MS)-based untargeted metabolomics approach to identify a wide range of endometrial metabolites for exploring the metabolic profiles of both two groups and their overlap.

## 2. Results

### 2.1. Clinical Evaluation of Dogs

The age, somatotype and breed of the dog population, i.e., 15 sick dogs with pyometra and 15 matched controls were taken into consideration for this study. As depicted in Table 1, the average age of sick dogs and control subjects were 9.33 ± 3.39 and 8.47 ± 2.67 (mean ± standard deviation, M ± SD) years, respectively, and the dogs were mainly of a small size in somatotype and purebred species in breed. There were no statistically significant differences in mean age, somatotype and breed for the healthy and diseased groups.

We performed blood tests for the diseased dogs with heathy dogs as controls. As expected, blood cell counts revealed significantly increased numbers of white blood cells (WBC) and neutrophils (Neu) in sick dogs with pyometra compared with healthy dogs (Table 2). By biochemistry analysis of the blood, the blood urea nitrogen (BUN) and globulin (GLO) contents were also elevated in the dogs that suffered pyometra (Table 2). In addition, an elevated level of CPR was observed in sick dogs using the CPR examination when compared to the controls (Table 2).

### 2.2. Ultrasonography and Histopathology

**Figure 1:**
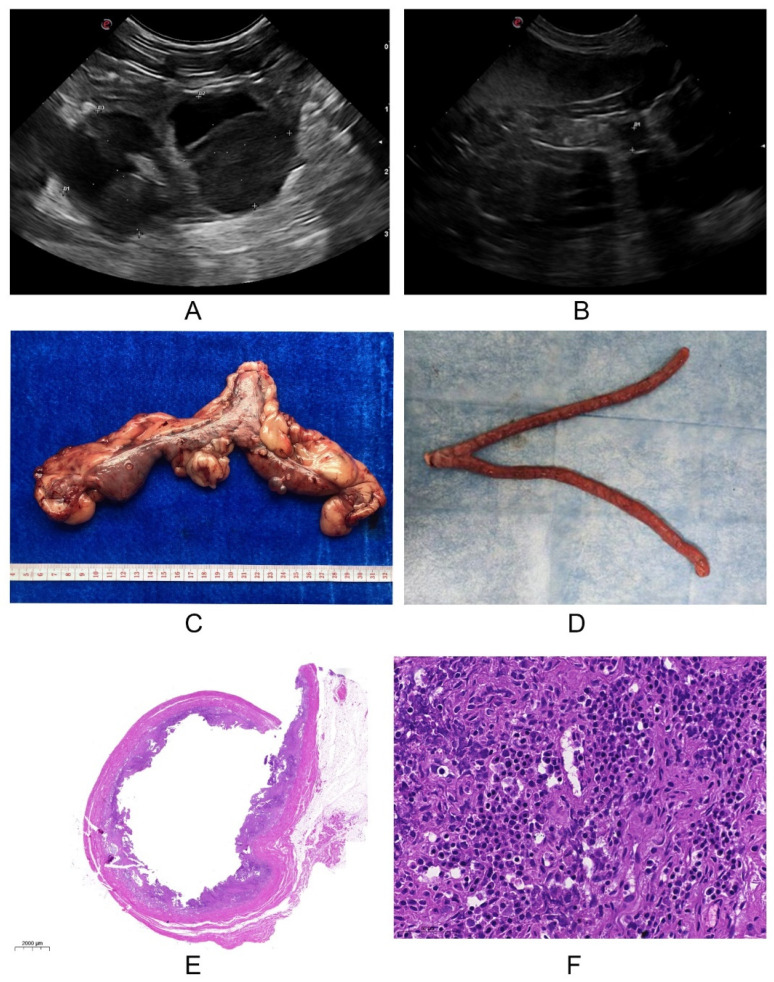
The clinical examinations of dogs. (**A**,**B**). Ultrasonography examination: the irregular and hypertrophic endometrium and fluid-filled uterine body were observed in dogs with pyometra (**A**) compared with that in healthy dogs (**B**); (**C**,**D**). The uterine examination: the fluid filled in the uteruses of dogs suffering from pyometra (**C**), but not in the healthy dogs (**D**); (**E**,**F**). Histopathological examination: severe endometrial gland hyperplasia (**E**) and the inflammatory cells (lymphocytes, plasma cells) endometrial infiltration (**F**) presented in the diseased group.

As a useful and reliable clinical diagnostic tool of canine pyometra, uterine ultrasonography found the presence of irregular and hypertrophic endometrium and fluid-filled uterine body in sick dogs with pyometra (Figure 1A), whereas these changes were not observed in healthy dogs (Figure 1B). Consistent with this, these changes could be directly observed in the excisional uteruses bodies of dogs suffering from pyometra (Figure 1C), but not in the control group (Figure 1D). These results were also confirmed histopathologically, which was characterized by the inflammatory cells (lymphocytes, plasma cells) endometrial infiltration and severe endometrial gland hyperplasia in the diseased group (Figure 1E,F) compared with that of healthy dogs.

### 2.3. Quality Control of Untargeted Metabolic Profiling

**Figure 2:**
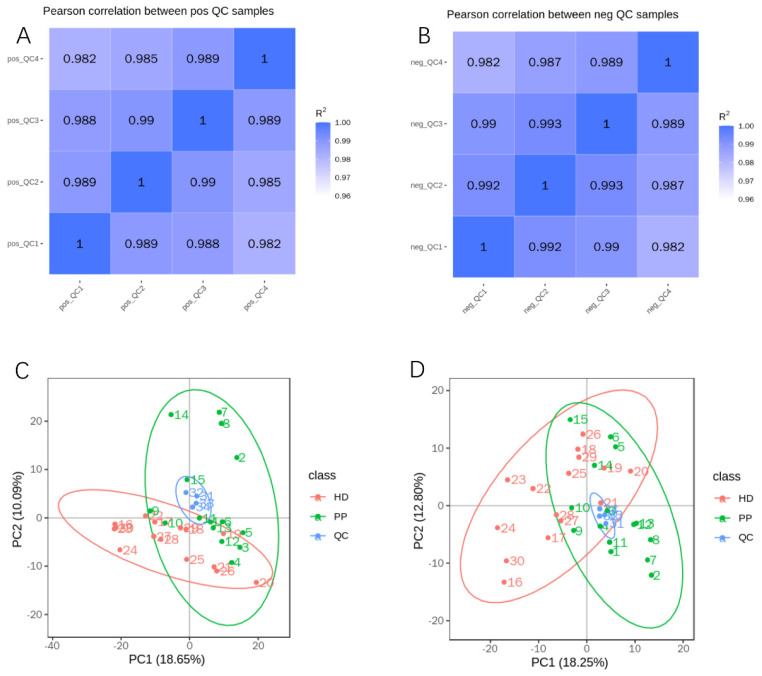
The quality control of untargeted metabolic profiling. (**A**,**B**). Pearson correlation analysis between QC samples: the coefficient (R2) values were both nearly 1 under the positive (**A**) or negative (**B**) polarity modes; (**C**,**D**). PCA analysis: QCs tightly aggregated into a cluster in the positive (**C**) and negative (**D**) ion modes.

The metabolome is easily interfered with by external factors, and it is very important to determine whether the instrument is stable and the signal response intensity is normal in the process of metabolite detection. To ensure the reliability of data, quality control (QC) was performed by the Pearson correlation analysis between QC samples and principal component analysis (PCA). By calculating the Pearson correlation coefficient (R2) between QC samples, we found that R2 values were both nearly 1 under the positive or negative polarity mode (Figure 2A,B), suggesting that the whole detection process had quite good stability and the data had a high quality. Moreover, PCA analysis showed that the QCs tightly aggregated into a cluster, which proved that there was great repeatability and stability of the analytical instrument (Figure 2C,D).

### 2.4. Untargeted Metabolic Profiling of Endometrium in Dogs with Pyometra

To achieve a comprehensive identification of canine endometrium metabolites, an untargeted metabolic profile was performed using Vanquish ultra-high performance liquid chromatography (UHPLC) system on the 30 endometrium samples, which commonly generated 705 ion features in the positive polarity mode and 414 ion features in the negative polarity mode. After integrating the retention time, exact mass and mass spectrometry (MS)/MS fragment, the metabolites were identified by comparing MS1 and MS2 data with those of corresponding compounds in public MS databases such as mz Cloud, mzVault, Mass List, Kyoto Encyclopedia of Genes and Genomes (KEGG), Human Metabolome Database (HMDB) and LIPID Maps. Lastly, a total of 275 differential metabolites (173 in positive and 102 in negative polarity modes) were commonly characterized in the PP group.

### 2.5. Screening of Differential Metabolites of Endometrium in Dogs with Pyometra

To better obtain the changes in different metabolites of endometrium in dogs with pyometra, a multivariate statistical analysis such as PCA and partial least squares discrimination analysis (PLS-DA), was applied and the relationship between biological characteristics and metabolomics was explored. Unsupervised PCA is a common pattern recognition method that was used to analyze the overall distribution of endometrium in the PP group, and to eliminate discrete abnormal data points. As shown in PCA score plots (Supplementary Figure S1), two groups were distinguished based on the first two principal components (PC1 and PC2), and metabolite profile differences were observed between the PP and HD groups in positive and negative modes.

**Figure 3:**
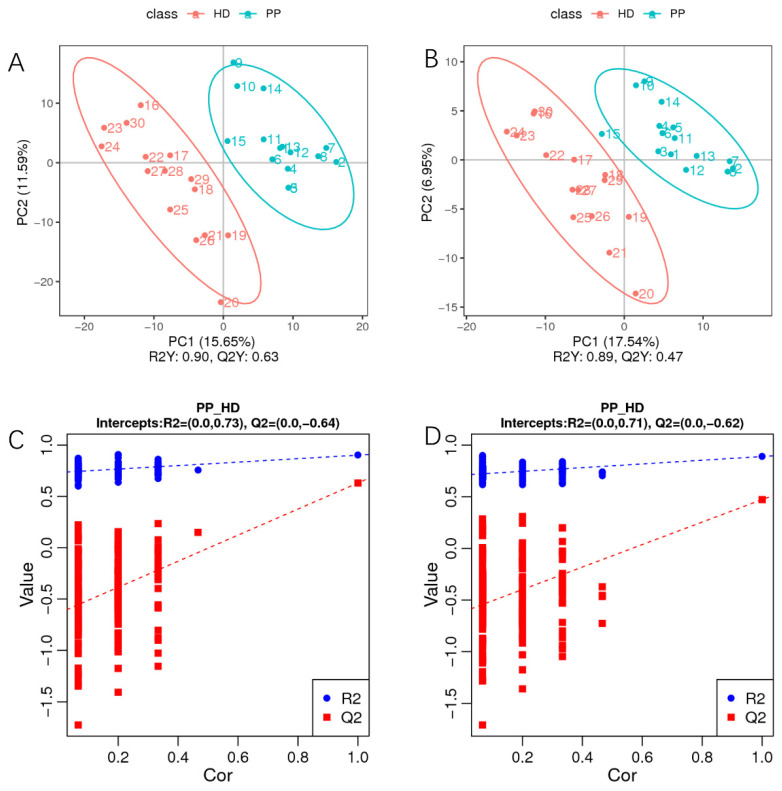
The partial least squares discrimination analysis (PLS-DA) of differential metabolites of endometrium in dogs with pyometra and healthy dogs. (**A**,**B**). The scattered plot: the classification parameters (R2Y) were 0.90 and 0.89 in positive (**A**) and negative (**B**) ionization modes; (**C**,**D**). The sorting inspection plot: the intercept values of R2 and Q2 were (0.0, 0.73) and (0.0, −0.64) in positive mode (**C**) and (0.0, 0.71) and (0.0, −0.62) in negative mode (**D**).

In addition, a supervised PLS-DA model was established to analyze the variables that contribute to sample classification in more detail. As displayed in Figure 3A,B, the classification parameters (R2Y) were 0.90 and 0.89 in positive and negative ionization modes, respectively. Furthermore, the Q2Y values were both 0.47 in positive and negative ion modes, indicating that there was good fitting and predictive power. A permutation test of 200 iterations showed that the intercept values of R2 and Q2 were (0.0, 0.73) and (0.0, −0.64) in positive mode (Figure 3C) and (0.0, 0.71) and (0.0, −0.62) in negative mode (Figure 3D), thus indicating that the PLS-DA model was reliable. Afterward, the variable importance in the projection (VIP) of the PLS-DA was used to determine the discriminating metabolites between the two group samples. In this study, metabolites with VIP > 1 or FC < 1.5 and *p*-value < 0.05 (ANOVA) were regarded as differential metabolites.

For the above statistical analysis, 1,119 critical metabolites determined in the positive (Supplementary Table S1) and negative ion modes (Supplementary Table S2) were responsible for the metabolic changes between the PP and HD groups.

### 2.6. Analysis of Differential Metabolites

**Figure 4:**
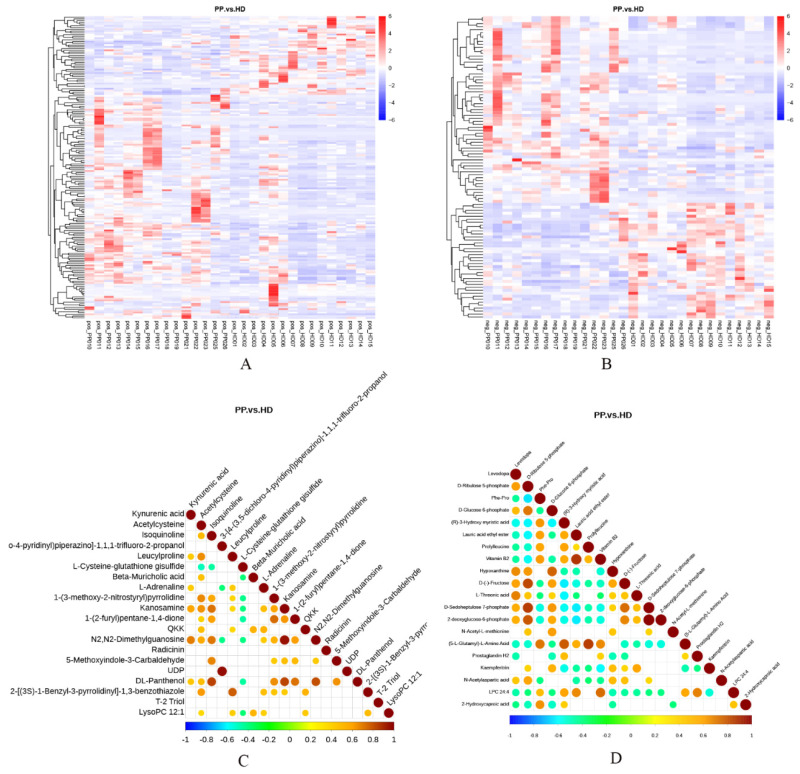
Correlation analysis of differential metabolites of endometrial tissues between dogs with pyometra and healthy dogs. (**A**,**B**). Heat map visualization of differential metabolites in the positive (**A**) and negative (**B**) modes; (**C**,**D**). Correlation diagram of differential metabolites in the positive (**C**) and negative (**D**) modes.

The heat maps of the canine endometrium samples versus differential metabolites under positive and negative ionization modes were revealed in Figure 4A,B. Clustering analysis based on the average algorithm indicated that the samples can be separated into two clusters, and the metabolites were significantly different between two groups. To determine the consistency of the changing trend of metabolites and metabolites, the correlation of each metabolite was analyzed by calculating the Pearson correlation coefficient among all metabolites, and a correlation diagram of differential metabolites was generated when there was a significant linear relationship between the two metabolites (*p* < 0.05) (Figure 4C,D). It was seen that Kynurenic acid had a significant synergistic effect with Leucylproline, L-Adrenaline, Kanosamine, N2, N2-Dimethylguanosine and DL-Panthenol on the positive ionization mode, and had a significantly mutually exclusive relation with Phe-Pro, (R)-3-Hydroxy myristic acid, Lauric acid ethyl ester, Prolylleucine, Vitamin B2, (5-L-Glutamyl)-L-Amino Acid, Prostaglandin H2, LPC 24:4 and 2-Hydroxycaproic acid on the negative ionization mode.

### 2.7. Metabolic Pathway Analysis

KEGG was a powerful tool for metabolism analysis and metabolic network research in organisms. Based on the content changes of differential endogenous metabolites of endometrium in the HD group and the reported pathway from the KEGG database, the overall pathways of endometrial metabolism in the PP group were obtained in the positive (Supplementary Table S3) and negative (Supplementary Table S4) ionization modes, illustrating that the diseased canine endometrium reacted to the changes in metabolites. Among them, Nicotinate and nicotinamide metabolism, Arachidonic acid metabolism, Serotonergic synapse and Tyrosine metabolism were considered to have a relationship with the occurrence of canine pyometra according to the number and role of differential metabolites.

**Figure 5:**
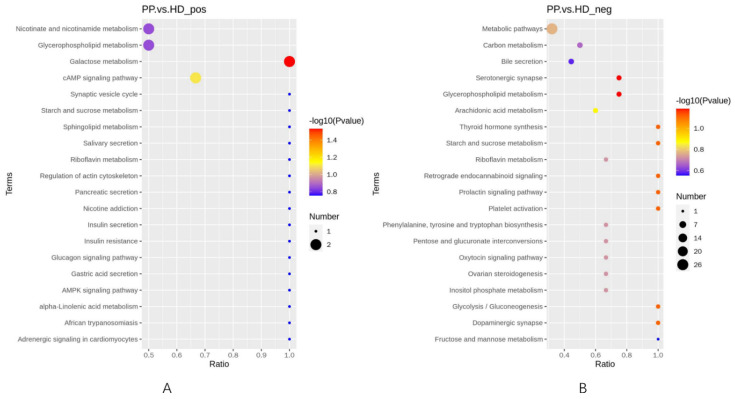
Significantly changed pathways based on the enrichment analysis. (**A**). KEGG enrichment bubble plot under the positive mode; (**B**). enrichment bubble plot under the negative mode.

To explore the important pathways related to the metabolic response to the diseased canine endometrium, the differential metabolites between the two group samples were subjected to pathway analysis using Metaboanalyst 4.0, which was shown in Figure 5. We found that some KEGG pathways enriched in the differential metabolites, including Galactose metabolism, cAMP signaling pathway and Glycerophospholipid metabolism, proving some insights into the metabolic changes during pyometra.

### 2.8. Receiver Operating Characteristic of Differential Metabolites Related to the Canine Pyometra

**Figure 6:**
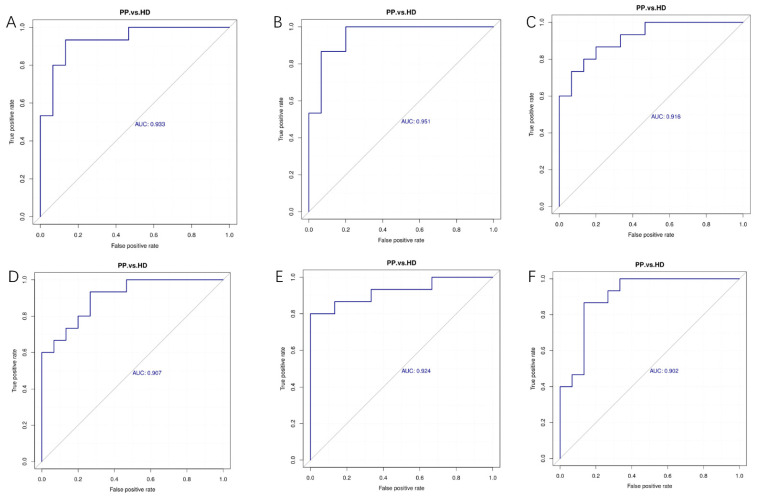
The receiver operating characteristic (ROC) curves analysis of differential metabolites. (**A**–**F**). AUC values of Tributyl citrate (**A**), Kynurenic acid (**B**), Acetylcysteine (**C**) and Isoquinoline (**D**) in the positive mode, and Phe-Pro (**E**), D-Glucose 6-phosphate (**F**) in the negative mode were all more than 0.9.

To evaluate the accuracy of differential metabolites as potential biomarkers of canine pyometra, the receiver operating characteristic (ROC) curves were drawn by a series of different dichotomies (cut-off value or decision threshold) based on the true positive rate (sensitivity) as the ordinate and the false positive rate (1-specificity) as the abscissa (Figure 6). In the ROC curves, its area below was called the area under the curve (AUC) which was used to estimate the sensitivity and specificity of biomarkers in predicting the occurrence of events, and the sensitivity and specificity of each metabolite were determined by the optimal threshold of the ROC curve. In general, the prediction of the occurrence of events have a low accuracy when AUC values are 0.5–0.7, and have a high accuracy when AUC values are more than 0.9. In this study, we observed that D-Ribulose 5-phosphate, Tributyl citrate, Kynurenic acid, Acetylcysteine, Isoquinoline, Phe-Pro, D-Glucose 6-phosphate and 3-[4-(3,5-dichloro-4-pyridinyl) piperazino]-1,1,1-trifluoro-2-propanol were able to be the potential biomarkers of canine pyometra.

## 3. Discussion

Pyometra is a common inflammatory disease of the uterus in sexually mature female dogs that frequently progresses into sepsis, and systemic inflammatory response syndrome (SIRS) [1,25]. Regarding its clinical diagnosis, ultrasonography and histopathology of the uterus were used to determine canine pyometra, with a clinical examination of systemic inflammation of dogs by hematology [26]. In our study, we summarized that pyometra mainly occurred in the small somatotype and old dogs, with a mean age of 9.33 ± 3.39 (M ± SD) years, and it was characterized by the irregular and hypertrophic endometrium and fluid-filled uterine body, which were confirmed by the histopathology of canine uteruses. Additionally, the blood examination found an increase in inflammatory cells (white blood cells and neutrophils) and C-reactive protein, suggesting that sick dogs with pyometra suffered from systemic inflammation, as described previously [25,27,28,29].

In terms of its treatment, OHE can effectively treat canine pyometra, but there is a serious disadvantage that an elevated risk of some diseases is observed after surgical operation, implying the importance of developing its early diagnosis and new treatment [7,8,9,10,11,12]. Generally, biomarkers play a significant role in the early diagnosis of diseases, and metabolite biomarkers are extensively applied in reproductive system diseases in humans, including endometriosis and endometrial cancer [19,23,24,30]. For defining the endometrial metabolites of dogs with pyometra, the endometrium tissues of sick dogs with pyometra and these controls were collected and detected utilizing a UHPLC-qTOF-MS-based untargeted metabolomics technology in this study. The great quality of data generated by this whole detection process was proved by the Pearson correlation analysis between QC samples and PCA, and this method was consistent with previous studies [31,32]. A total of 705 ion features in the positive polarity mode and 414 ion features in the negative polarity mode were identified in the endometrium tissues of dogs. After function and annotation by comparison with public MS databases such as KEGG, HMDB and LIPID Maps, we found that these metabolites mainly participated in the lipid metabolism, global and overview maps, and amino acid metabolism, and the metabolites mainly were lipids and lipid-like molecules, organic acids and derivatives, and organic oxygen compounds. Subsequently, a lipid category of metabolites was conducted, and a total of 176 metabolites were lipids, with 10 eicosanoids [FA03], 10 glycerophosphocholines [GP01], 13 glycerophosphoethanolamines [GP02], 12 sterols [ST05], 26 fatty acids and conjugates [FA01] and 24 glycerophosphocholines [GP01]. These results suggested that metabolites related to canine pyometra might participate in lipid metabolism.

Moreover, 173 ion features in the positive polarity mode and 102 ion features in the negative polarity mode showed significant differences between the two groups by the multivariate statistical analysis of unsupervised PCA and the supervised PLS-DA model. On the differential metabolites, a total of 180 metabolites were significantly upregulated in dogs with pyometra, with 95 downregulated metabolites. On the upregulated metabolites, kynurenic acid is the major metabolite in the tryptophan metabolism pathway which involved many diseases of the peripheral nervous system and central nervous system, including cancers, inflammatory diseases, nervous system diseases, mental diseases and neurodegenerative diseases [33], and the infection and inflammation could induce and activate some enzymes in kynurenine pathway [34]. It implied that the change of kynurenic acid may be associated with the occurrence of canine pyometra. The other upregulated metabolites of endometrium in sick dogs with pyometra, isoquinoline and acetylcysteine had pharmacological features and multi-target potential for complex diseases [35,36]. On the downregulated metabolites, estrone is an estrogen secreted by humans and animals, and can decrease the risk rate of pyometra [7,37]. In accordance with this, we also observed the downregulation of estrone in metabolite of endometrium in sick dogs with pyometra compared with that of healthy dogs, which again indicated that estrone is associated with the occurrence of pyometra, and again confirmed the accuracy of data in this study. Moreover, β-Nicotinamide mononucleotide is a key intermediate of an essential coenzyme for cellular redox reactions, nicotinamide adenine dinucleotide (NAD), which can improve various symptoms, such as diabetes and age-related physiological decline [38]. Interestingly, we found that it also had significant downregulation in the endometrium in sick dogs with pyometra, but the role of β-Nicotinamide mononucleotide on canine pyometra is necessary to further verify. Finally, kynurenic acid is expected to become a biomarker of pyometra in dogs, and is conducive to the early diagnosis of the disease.

## 4. Materials and Methods

### 4.1. Animals and Samplings

Voluntary dog owners were recruited for the canine uterus donation, and the uterus collection was conducted from 30 privately-owned dogs with the owner’s written consent in Changchun of Jilin province, China, from March 2021 until February 2022. Among them, the healthy dogs (n = 15) referred no disease, and the remaining dogs (n = 15) were suspected of suffering from pyometra; during this period, these dogs were not subjected to any drug treatment according to their owners (Supplementary Table S5). All dogs were subjected to ovariohysterectomy after imaging procedures.

The acquisition steps of endometrial tissues were as follows: after an operation, the uterus was rapidly moved to an ultra-clean cabinet for washing with saline and disinfecting with 75% ethanol, and then the endometrial tissues were harvested and stored in a −80 °C refrigerator (TSX70086V, Thermo Scientific, Waltham, MA, USA) prior to metabolomics analysis. In addition, every uterine tissue was fixed in 10% neutral buffered formalin for histological examination, and blood samples were also collected from every dog.

### 4.2. Ultrasonography Examinations

Owners were asked to complete a preliminary questionnaire to ensure the basic information about dogs, and the brief description was related to the dog’s age, breed and health status. The belly of the dogs was directly examined by ultrasonography to evaluate uterine lesions, and its examination steps were as follows: firstly, the dogs were gently restrained in the U-shaped slot in a supine position without the use of sedative drugs, and then were directly smeared medical coupling gel in the skin after abdominal shaving; subsequently, the scanning probe (Esoate, Firenze, Italy) was put in the space between the front edge of the pubic bone and the posterior part of bilateral kidneys to detect the uterus.

### 4.3. Blood Examinations

To further the inflammatory status of diseased dogs with pyometra, blood examinations were conducted in this study, including the blood routine, blood biochemical examination and C-reactive protein. For the blood routine, 2 mL of blood samples were collected from the small saphenous vein on the lateral side of the hind limb of every dog into a sterile vacutainer tube which contained the Ethylene Diamine Tetraacetic Acid (EDTA), and then was estimated their complete blood counts using a BC-5000 Vet Hematology Analyzer (Mindray, Shenzhen, China). For other blood tests, 2 mL of blood samples collected in the same way was centrifugated at 3500× *g* for 10 min at 4 °C to harvest the serum, which enabled the serum biochemical analyses and CPR.

### 4.4. Histopathology Examinations

For the further identification of canine pyometra, the hematoxylin-eosin (HE) staining was conducted in this study. Firstly, the partial uterus of every dog was prefixed in 10% neutral formalin solution for 24 h after OHE, and then dehydrated in a graded series of increasing concentrations of ethanol (70–100%) and put in xylene to wash the residual alcohol. After their deparaffinization and rehydration, 5 μm longitudinal sections were stained with hematoxylin solution for 3 min followed by being soaked for 30 s in 1% acid ethanol and then rinsed for 1 min in distilled water. Next, the sections were stained with eosin counterstain for 3 min and followed by dehydration with graded alcohol (80%, 95%, 95%, 95%, 100%, 100%, 100%) and immersion with xylene.

### 4.5. Metabolites Extraction

A total of 100 mg canine endometrial tissues were individually grounded into power under liquid nitrogen, with resuspension in 500 μL of prechilled 80% methanol by a vortex oscillator. Subsequently, these samples were stewed on ice for 5 min and centrifuged for 20 min at 15,000× *g* at 4 °C to gain the supernatant, and then were diluted to a final concentration containing 53% methanol by LC-MS grade water, which was again centrifuged in the same condition. Lastly, the obtained supernatant was injected into the Liquid chromatograph-mass spectrometer/Mass Spectrometry (LC-MS/MS) system analysis [39].

### 4.6. UHPLC-MS/MS Analysis

UHPLC-MS/MS analysis was carried out by a Vanquish UHPLC system (Thermo Fisher, Berlin, Germany) and an Orbitrap Q ExactiveTM HF mass spectrometer (Thermo Fisher, Berlin, Germany) in Novogene Co., Ltd. (Beijing, China). Canine endometrial samples were injected into a Hypesil Gold column (C18, 100 × 2.1 mm, 1.9 μm) at a flow rate of 0.2 mL/min using a 17 min linear gradient. The eluents for the positive polarity mode were eluent A of 0.1% formic acid and eluent B of methanol, whereas the eluents A and B for the negative polarity mode were 5 mM ammonium acetate (pH 9.0) and methanol, respectively. The chromatographic gradient elution procedure was as follows: 2% B for 1.5 min, 2–100% B for 3 min, 100% B for 10 min, 100–2% B for 10.1 min and 2% B for 12 min. The Q Exactive TM HF mass spectrometer was working under positive/negative polarity mode, and the parameters were set, as follows: spray voltage of 3.5 kilovolts (kV), sheath gas flow rate of 35-pound square inch (psi), an auxiliary gas flow rate of 10 L/min, the capillary temperature of 320 °C, S-lens RF level of 60 and auxiliary gas heater temperature of 350 °C.

### 4.7. Data Processing and Metabolite Identification

The raw data files of canine endometrial samples were obtained by UHPLC-MS/MS and further handled using the Compound Discoverer 3.1 (CD3.1, Thermo Fisher) for performing the peak alignment, peak picking and quantitation of every metabolite. The major parameters concerned in this research contained 12 s retention time tolerance, 5 part per million (ppm) actual mass tolerance, 30% signal intensity tolerance, 3 signal-to-noise ratio and the minimum signal intensity. Meanwhile, the peak intensities were normalized to the total spectral intensity, and then were for predicting the molecular formula on the basis of additive ions, molecular ion peaks and fragment ions. After that, the accurate qualitative and relative quantitative results were generated by matching these peaks with the mzCloud (accessed on 13 February 2021, https://www.mzcloud.org/), mzVault and MassList databases. Statistical analysis was determined by the operating system CentOS (version 6.6), software R (version 3.4.3) and Python (version 2.7.6).

### 4.8. Data Analysis

The metabolites annotation of canine endometrial samples was performed on the KEGG database (accessed on 13 February 2021, https://www.genome.jp/kegg/pathway.html), HMDB database (accessed on 13 February 2021, https://hmdb.ca/metabolites) and LIPID Maps database (accessed on 13 February 2021, http://www.lipidmaps.org/). For the multivariate statistical analysis, a complete data processing software metaX [40] was used for PCA and PLS-DA after the data conversion to get the VIP value of each metabolite. For the univariate analysis, we applied the *t*-test to calculate the statistical significance (*p*-value) and the fold change (FC-value) of both metabolites in the two groups. The screening standard of differential metabolites was VIP (variable importance in the projection) > 1 and *p* < 0.05 and FC ≥ 2 or FC ≤ 0.5. Volcano plots were drawn by ggplot2 in R language, which could choose important metabolites using the VIP, log2 (FC-value) and −lg (*p*-value) of metabolites. The clustering heat maps were generated by the Pheatmap package in R language, and these data were normalized by z-scores of the intensity areas of differential metabolites in both endometrial samples of healthy dogs and diseased dogs with pyometra. Additionally, the correlation analysis (Pearson correlation coefficient) and plots between differential metabolites of two groups were respectively conducted using the cor of R language and a corrplot package in R language, and their significant differences were calculated by the cor.mtest, which was considered as a statistical difference with *p* < 0.05. Moreover, the bubble chart was plotted using ggplot2 in R language, and the functions of these metabolites and metabolic pathways were researched by the KEGG database in this study. The metabolic pathways enrichment of differential metabolites was also carried out, which was considered as the enrichment of metabolic pathway when the ratio of x/n was higher than that of y/n, and a metabolic pathway was supposed to have a statistically significant enrichment when its *p*-value was less than 0.05.

## 5. Conclusions

Taken together, in the present study, canine pyometra was firstly identified by a combination of clinical and laboratory examinations, and then a wide range of endometrial metabolites of healthy dogs and diseased dogs with pyometra were defined utilizing a UHPLC-qTOF-MS-based untargeted metabolomics approach. By the multivariate statistical analysis, the endometrial metabolites of the two groups had a significant difference, and kynurenic acid was expected to become a biomarker of pyometra in dogs, which provided a new idea for exploring the safe and effective therapy in the treatment of canine pyometra.

## Figures and Tables

**Table 1:** The clinical information of healthy dogs and diseased dogs with pyometra.

Items	Dogs with Pyometra	Healthy Dogs
Mean age (years) ± SD	9.33 ± 3.39	8.47 ± 2.67
Number of breeds		
Teddy dog	5	7
Poodle	2	1
Border Collie	1	2
Pomeranian	1	3
Other	6	2
Number of somatotypes		
Large	2	0
Medium	2	3
Small	11	12
Number of lineages		
Mix breed	0	0
Pure breed	15	15

**Table 2:** The blood examinations of diseased dogs with pyometra and healthy dogs.

Items	Dogs with Pyometra			Healthy Dogs			Reference	Unit	*p*-Value
	Min	Max	Mean	Min	Max	Mean			
Blood routine									
White blood cell (WBC)	7.13	48.23	19.25 ± 12.70	6.92	13.96	9.61 ± 2.00	6.00–17.00	10^9^/L	0.007
Neutrophils (Neu)	4.79	44.64	16.33 ± 11.73	4.53	9.11	6.46 ± 1.56	3.62–12.30	10^9^/L	0.003
Blood biochemical examination									
Blood urea nitrogen (BUN)	3.43	15.90	9.67 ± 4.02	2.98	6.94	5.22 ± 1.11	2.5–9.6	mmol/L	0.0002
Globulins (GLO)	29.80	67.20	52.05 ± 11.86	25.2	40.8	33.87 ± 4.43	23–52	g/L	5.97 × 10^−6^
C-reactive protein									
c-CRP	11.00	47.10	22.20 ± 11.22	1.1	9.9	4.33 ± 2.34	16–218	mg/L	3.48 × 10^−6^

## Data Availability

The metabolomics datasets can also be accessed at MetaboLights [https://www.ebi.ac.uk/metabolights/], accessed on 13 February 2021, (Project ID: MTBLS6165).

## Supplementary Materials

The following supporting information can be downloaded at: https://www.mdpi.com/article/10.3390/ijms232214161/s1, Figure S1. PCA score plots of differential metabolites of endometrium in dogs with pyometra and healthy dogs.; Table S1. Differential metabolites identified in positive ion mode of endometrium of diseased and healthy dogs.; Table S2. Differential metabolites identified in negative ion mode of endometrium of diseased and healthy dogs.; Table S3. The enriched pathways of differential endometrial metabolism in the positive ionization mode.; Table S4. The enriched pathways of differential endometrial metabolism in the negative ionization mode.; Table S5. The clinical information of diseased dogs with pyometra and healthy dogs.
