# Supplementary material for: Identification of Canine Pyometra-Associated Metabolites Using Untargeted Metabolomics
Source: Int J Mol Sci. 2022 Nov 16;23(22):14161. doi: 10.3390/ijms232214161 (PMC9697130; doi:10.3390/ijms232214161)
Supplement: Supplementary file 1 [file ijms-23-14161-s001.zip › Figure S1.pdf]

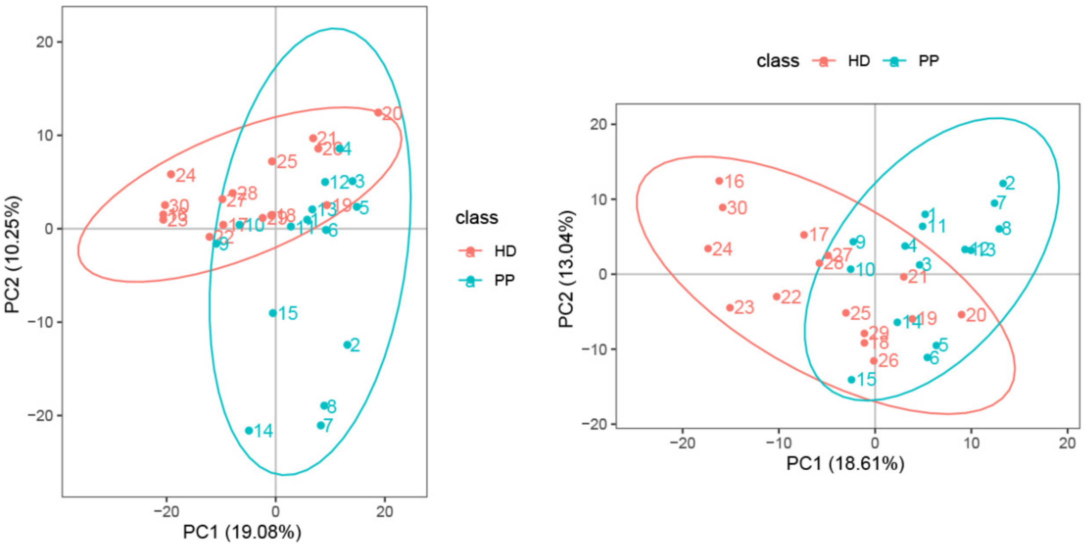

A B

Figure S1 PCA score plots of differential metabolites of endometrium in dogs with pyometra and healthy dogs. A. PCA score plots of differential metabolites of endometrium under the positive ionization mode; B. PCA score plots of differential metabolites of endometrium under the negative ionization mode.
