# Supplementary material for: Identification of Canine Pyometra-Associated Metabolites Using Untargeted Metabolomics
Source: Int J Mol Sci. 2022 Nov 16;23(22):14161. doi: 10.3390/ijms232214161 (PMC9697130; doi:10.3390/ijms232214161)
Supplement: Supplementary file 1 [file ijms-23-14161-s001.zip › Table S1.pdf]

Table S1. Differential metabolites identified in positive ion mode of endometrium of diseased and healthy dogs.

Name	Formula	Molecular Weight	RT [min]	FC	log2FC	P value	ROC	VIP	Up/ Down
Kynurenic acid	C10H7NO3	189.04	5.37	8.27	3.05	0.00	0.95	2.30	up
Acetylcysteine	C5H9NO3S	163.03	1.39	2.54	1.34	0.00	0.92	2.07	up
Isoquinoline	C9H7N	129.06	5.58	3.51	1.81	0.00	0.91	1.99	up
3-[4-(3,5-dichloro-4-pyridinyl)piperazino]-1,1,1-trifluoro-2-propanol	C12H14Cl2F3N3O	343.05	2.44	0.29	-1.77	0.00	0.93	2.01	down
Leucylproline	C11H20N2O3	228.15	5.03	5.69	2.51	0.00	0.89	1.91	up
L-Cysteine-glutathione gisulfide	C13H22N4O8S2	426.09	1.36	0.54	-0.88	0.00	0.91	2.02	down
Beta-Muricholic acid	C24H40O5	408.29	7.45	8.78	3.13	0.00	0.88	2.09	up
L-Adrenaline	C9H13NO3	183.09	5.02	3.38	1.76	0.00	0.85	2.02	up
1-(3-methoxy-2-nitrostyryl)pyrrolidine	C13H16N2O3	496.23	4.79	4.60	2.20	0.00	0.90	1.91	up
Kanosamine	C6H13NO5	179.08	4.94	2.61	1.38	0.00	0.88	1.87	up
1-(2-furyl)pentane-1,4-dione	C9H10O3	188.05	5.66	9.42	3.24	0.00	0.90	1.93	up
QKK	C17H34N6O5	385.23	4.98	2.29	1.19	0.00	0.83	1.86	up
N2,N2-Dimethylguanosine	C12H17N5O5	311.12	4.94	2.45	1.29	0.00	0.86	1.81	up
Radicinin	C12H12O5	258.05	5.79	0.22	-2.20	0.00	0.89	2.01	down
5-Methoxyindole-3-Carbaldehyde	C10H9NO2	175.06	5.69	1.68	0.75	0.00	0.87	1.79	up
UDP	C9H14N2O12P2	404.00	1.82	0.25	-2.02	0.00	0.88	1.91	down
DL-Panthenol	C9H19NO4	205.13	1.38	3.67	1.87	0.00	0.84	1.77	up
2-[(3S)-1-Benzyl-3-pyrrolidinyl]-1,3-benzothiazole	C18H18N2S	294.12	4.92	2.09	1.06	0.00	0.86	1.79	up
T-2 Triol	C20H30O7	382.20	7.45	0.01	-6.65	0.00	0.89	1.95	down

LysoPC 12:1	C20H36NO7P	433.22	4.76	3.93	1.97	0.00	0.83	1.90	up
Biopterin	C9H11N5O3	237.09	6.07	0.52	-0.95	0.00	0.88	1.77	down
Octyl hydrogen phthalate	C16H22O4	256.17	6.72	2.09	1.06	0.00	0.88	1.74	up
Valylproline	C10H18N2O3	428.26	4.77	2.41	1.27	0.00	0.84	1.73	up
D-3-Phenyllactic acid	C9H10O3	166.06	5.67	4.49	2.17	0.00	0.88	1.79	up
SDB-006 N-phenyl analog	C20H22N2O	612.35	4.67	3.32	1.73	0.00	0.82	1.73	up
Tributyl citrate	C18H32O7	360.21	7.45	0.02	-5.95	0.00	0.93	1.88	down
2-(2-amino-3-methylbutanamido)-3-phenylpropanoic acid	C14H20N2O3	264.15	5.27	2.27	1.18	0.00	0.83	1.73	up
4-chloro-5-morpholino-2-quinoxalin-2-ylpyridazin-3(2H)-one	C16H14ClN5O2	343.08	2.01	0.48	-1.05	0.00	0.84	1.67	down
D-Fructose 6-phosphate	C6H13O9P	260.03	1.31	0.40	-1.34	0.00	0.83	1.64	down
KLK	C18H37N5O4	369.27	9.99	5.06	2.34	0.00	0.83	1.65	up
DL-Carnitine	C7H15NO3	161.11	1.43	2.99	1.58	0.00	0.82	1.65	up
Asp-Phe	C13H16N2O5	280.11	5.02	1.72	0.78	0.00	0.83	1.66	up
Isobutyryl carnitine	C11H21NO4	231.15	5.15	4.42	2.14	0.00	0.82	1.62	up
gamma-Glutamylglutamine	C10H17N3O6	275.11	1.37	3.35	1.75	0.00	0.81	1.61	up
L(-)-Carnitine	C7H15NO3	161.11	1.29	2.64	1.40	0.00	0.82	1.59	up
4-(4-methoxyphenyl)-6-pyridin-4-yl-1,3,5-triazin-2(3H)-one	C15H12N4O2	318.05	6.04	0.57	-0.82	0.00	0.83	1.58	down
3-(3,4,5-trimethoxyphenyl)propanoic acid	C12H16O5	222.09	5.89	2.27	1.18	0.00	0.84	1.60	up
6-methyl-4-(morpholinomethyl)-2H-chromen-2-one	C15H17NO3	259.12	6.13	0.53	-0.91	0.00	0.80	1.60	down
5α-Dihydrotestosterone glucuronide	C25H38O8	466.25	6.06	0.56	-0.84	0.00	0.79	1.61	down
Glycyl-L-leucine	C8H16N2O3	188.12	4.68	3.17	1.67	0.00	0.79	1.60	up

Gelsemine	C20H22N2O2	322.16	5.25	66.72	6.06	0.00	0.84	1.62	up
Thr-Leu	C10H20N2O4	232.14	5.07	1.64	0.71	0.00	0.80	1.60	up
2,4-Dimethylbenzaldehyde	C9H10O	134.07	6.35	1.52	0.60	0.00	0.84	1.54	up
PC (22:6e/5:0)	C35H60NO7P	637.40	9.27	0.56	-0.82	0.00	0.83	1.54	down
Acetylcholine	C7H15NO2	145.11	1.34	0.56	-0.85	0.00	0.81	1.62	down
8,15-Dihete	C20H32O4	336.23	5.97	0.43	-1.21	0.00	0.79	1.66	down
MNK	C15H29N5O5S	391.19	5.12	0.41	-1.28	0.00	0.79	1.88	down
SPH	C14H21N5O5	321.14	5.04	0.36	-1.48	0.00	0.82	1.56	down
Deoxyadenosine	C10H13N5O3	251.10	1.48	0.65	-0.63	0.00	0.81	1.55	down
N-Acetyl-L-leucine	C8H15NO3	173.11	5.61	2.19	1.13	0.00	0.80	1.58	up
2-methyl-2,3,4,5-tetrahydro-1,5-benzoxazepin-4-one	C10H11NO2	159.07	5.15	1.69	0.75	0.00	0.79	1.58	up
Gly-Tyr	C11H14N2O4	238.09	3.01	1.69	0.76	0.00	0.78	1.59	up
L-Threonic acid-1,4-lactone	C4H6O4	118.03	1.97	0.66	-0.61	0.00	0.80	1.54	down
HPH	C17H23N7O4	778.36	4.89	2.04	1.03	0.00	0.78	1.50	up
5-(2,5-dihydroxyhexyl)oxolan-2-one	C10H18O4	224.10	5.69	1.90	0.92	0.00	0.82	1.52	up
N1-(1H-indol-4-yl)cyclohexane-1-carboxamide	C15H18N2O	242.14	6.99	0.44	-1.19	0.00	0.82	1.60	down
α-Aspartylphenylalanine	C13H16N2O5	280.11	5.20	4.01	2.00	0.00	0.78	1.52	up
Riboflavin	C17H20N4O6	376.14	5.32	2.77	1.47	0.00	0.79	1.51	up
Ala-Leu	C9H18N2O3	202.13	4.92	1.71	0.78	0.00	0.80	1.62	up
PS (19:2/19:2)	C44H78NO10P	811.54	11.77	0.34	-1.55	0.00	0.80	1.51	down
LQH	C17H28N6O5	396.22	5.63	0.50	-1.01	0.00	0.77	1.71	down
5,6-dihydroxyindole	C8H7NO2	149.05	4.74	1.66	0.73	0.00	0.78	1.49	up
2-(acetylamino)-3-(1H-indol-3-yl)propanoic acid	C13H14N2O3	246.10	5.57	1.87	0.90	0.00	0.77	1.47	up

SMK	C14H28N4O5S	324.18	5.17	1.58	0.66	0.01	0.80	1.56	up
PC (20:5e/4:0)	C32H56NO7P	597.38	9.61	2.61	1.38	0.01	0.78	1.48	up
N3,N4-Dimethyl-L-arginine	C8H18N4O2	202.14	1.55	2.19	1.13	0.01	0.81	1.50	up
ethyl 4-({[(3-morpholinopropyl)amino]carbothioyl}amino)benzoate	C17H25N3O3S	373.15	4.67	2.70	1.43	0.01	0.78	1.46	up
Hydrocortisone acetate	C23H32O6	404.22	9.32	1.51	0.60	0.01	0.80	1.46	up
3-(3,4-Dihydroxyphenyl)-2-Methylalanine	C10H13NO4	211.08	3.92	6.61	2.72	0.01	0.79	1.50	up
2-Aminoacetophenone	C8H9NO	135.07	4.85	2.08	1.06	0.01	0.80	1.45	up
4-Hydroxyisoleucine	C6H13NO3	147.09	1.40	1.82	0.86	0.01	0.81	1.46	up
4-hydroxy-5,8-dimethylquinoline-3-carboxylic acid	C12H11NO3	217.07	5.29	3.69	1.88	0.01	0.78	1.42	up
6-Keto-prostaglandin f1alpha	C20H34O6	370.24	5.94	0.65	-0.62	0.01	0.82	1.43	down
Gly-Val	C7H14N2O3	174.10	2.06	3.19	1.67	0.01	0.78	1.49	up
VLH	C17H29N5O4	367.22	5.55	0.61	-0.70	0.01	0.77	1.44	down
Leukotriene B4 Ethanolamide	C22H37NO4	401.25	8.83	2.43	1.28	0.01	0.84	1.55	up
6-Methylquinoline	C10H9N	143.07	5.15	1.64	0.72	0.01	0.77	1.46	up
N6,N6,N6-Trimethyl-L-lysine	C9H20N2O2	188.15	1.20	0.58	-0.79	0.01	0.74	1.44	down
XLR11 N-(2-fluoropentyl) isomer	C21H28FNO	329.22	5.59	2.89	1.53	0.01	0.76	1.41	up
2-[(3S)-1-(2-Methylbenzyl)-3-pyrrolidinyl]-1,3-benzoxazole	C19H20N2O	292.15	5.26	56.05	5.81	0.01	0.73	1.45	up
4-Guanidinobutyric acid	C5H11N3O2	145.09	1.72	58.54	5.87	0.01	0.77	1.41	up
ACar 12:3	C19H32NO4	337.22	5.84	2.48	1.31	0.01	0.83	1.40	up
Hypoglycin A	C7H11NO2	141.08	1.41	3.38	1.76	0.01	0.77	1.43	up
All-Trans-13,14-Dihydroretinol	C20H32O	288.25	9.49	5.74	2.52	0.01	0.81	1.40	up

1-Methyladenosine	C11H15N5O4	281.11	1.88	1.82	0.86	0.01	0.76	1.44	up
Tomatidine	C27H45NO2	415.35	5.89	0.37	-1.45	0.01	0.89	1.59	down
N1-(1,3-thiazol-2-yl)-2-(1-adamantyl)acetamide	C15H20N2OS	276.13	6.86	0.27	-1.88	0.01	0.88	1.62	down
Phosphoethanolamine	C2H8NO4P	141.02	1.31	0.62	-0.69	0.01	0.74	1.41	down
Norfenefrine	C8H11NO2	153.08	3.21	29.20	4.87	0.01	0.80	1.41	up
tert-Butyl N-[1-(aminocarbonyl)-3-methylbutyl]carbamate	C11H22N2O3	230.16	5.16	2.01	1.01	0.01	0.77	1.45	up
N-(4-chlorophenyl)-N'-(2-phenoxyphenyl)urea	C19H15ClN2O2	360.07	1.39	1.77	0.82	0.01	0.83	1.35	up
5-Methyl-dl-tryptophan	C12H14N2O2	218.11	5.29	2.09	1.06	0.01	0.76	1.36	up
Val-Ser	C8H16N2O4	204.11	2.46	1.89	0.92	0.01	0.77	1.48	up
N-Acetylmethionine	C7H13NO3S	191.06	5.26	1.63	0.70	0.01	0.71	1.38	up
Pyridoxine	C8H11NO3	169.07	5.01	1.62	0.70	0.01	0.76	1.36	up
Prostaglandin K2	C20H30O5	332.20	7.62	2.57	1.36	0.01	0.77	1.34	up
PC (18:3e/4:0)	C30H56NO7P	573.38	9.73	1.80	0.85	0.01	0.76	1.40	up
19-Nortestosterone	C18H26O2	274.19	5.18	7.24	2.86	0.01	0.73	1.39	up
Isopropyl myristate	C17H34O2	287.28	6.79	0.45	-1.15	0.01	0.86	1.57	down
UR-144 N-(2-hydroxypentyl) metabolite	C21H29NO2	327.22	5.28	1.59	0.67	0.01	0.78	1.39	up
LysoPC 18:0	C26H54NO7P	523.36	9.10	0.59	-0.76	0.01	0.77	1.39	down
gamma-Glutamylleucine	C11H20N2O5	260.14	5.32	1.80	0.85	0.01	0.74	1.36	up
Glycerol-3-phosphate	C3H9O6P	172.01	1.45	0.30	-1.73	0.01	0.76	1.31	down
Verapamil hydrochloride	C27H38N2O4	454.29	9.80	2.72	1.44	0.01	0.75	1.31	up
PC (20:3e/20:4)	C48H84NO7P	817.59	11.53	2.85	1.51	0.01	0.74	1.31	up
Hexanoylcarnitine	C13H25NO4	259.18	5.39	2.15	1.10	0.01	0.76	1.31	up

3-Methoxy prostaglandin F1α	C21H38O6	408.25	6.76	0.47	-1.08	0.01	0.78	1.34	down
PC (18:4e/6:0)	C32H58NO7P	599.40	10.19	1.72	0.78	0.01	0.75	1.36	up
FLK	C21H34N4O4	203.13	2.15	20.04	4.32	0.01	0.75	1.30	up
1-Methylhistidine	C7H11N3O2	169.09	1.27	2.76	1.47	0.01	0.76	1.31	up
WKK	C23H36N6O4	230.14	3.89	5.63	2.49	0.01	0.77	1.33	up
LPE 18:0	C23H48NO7P	481.32	9.98	1.91	0.93	0.01	0.76	1.35	up
Diacetoxyscirpenol	C19H26O7	348.16	5.58	0.49	-1.02	0.01	0.77	1.52	down
L-Argininosuccinate	C10H18N4O6	290.12	1.56	3.34	1.74	0.01	0.69	1.32	up
IRH	C18H32N8O4	446.24	5.79	0.48	-1.06	0.02	0.76	1.43	down
2-hydroxy-6-[(8Z,11Z)-pentadeca-8,11,14-trien-1-yl]benzoic acid	C22H30O3	342.22	7.90	0.49	-1.02	0.02	0.73	1.29	down
L-Homocitrulline	C7H15N3O3	189.11	1.39	3.43	1.78	0.02	0.75	1.32	up
H-Gly-Pro-OH	C7H12N2O3	172.08	1.39	3.43	1.78	0.02	0.75	1.31	up
L-Kynurenine	C10H12N2O3	208.08	4.76	1.98	0.98	0.02	0.76	1.27	up
SNK	C13H25N5O6	658.35	4.98	1.89	0.92	0.02	0.72	1.27	up
5-Hydroxyindole-3-acetic acid	C10H9NO3	191.06	4.76	1.98	0.98	0.02	0.76	1.26	up
MKK	C17H35N5O4S	405.24	5.57	0.54	-0.89	0.02	0.72	1.43	down
Glycoursodeoxycholic acid	C26H43NO5	449.31	7.33	0.45	-1.15	0.02	0.79	1.36	down
Sodium Dehydrocholate	C24H33NaO5	424.22	6.63	0.58	-0.80	0.02	0.77	1.25	down
2,4-dihydroxyheptadec-16-en-1-yl acetate	C19H36O4	368.26	6.64	0.57	-0.81	0.02	0.79	1.30	down
4-Hydroxypropranolol	C16H21NO3	275.15	1.32	1.68	0.75	0.02	0.78	1.27	up
ACar 20:4	C27H46NO4	447.33	7.05	1.88	0.91	0.02	0.76	1.29	up
13-HPODE	C18H32O4	312.23	8.00	2.78	1.47	0.02	0.69	1.22	up
Jasmonic acid	C12H18O3	210.13	7.04	0.51	-0.97	0.02	0.73	1.23	down
PE (4:0/16:4)	C25H42NO8P	515.27	6.98	0.49	-1.02	0.02	0.77	1.22	down

WLH	C23H30N6O4	436.23	5.35	0.26	-1.94	0.02	0.78	1.39	down
Nicotinamide N-oxide	C6H6N2O2	138.04	1.81	0.49	-1.02	0.02	0.72	1.35	down
3-N-Methyl-L-histidine	C7H13N3O3	187.10	1.68	4.67	2.22	0.02	0.75	1.31	up
methyl 2-[(2-acetyl-3-oxo-1-butenyl)amino]acetate	C9H13NO4	199.08	5.13	7.68	2.94	0.02	0.79	1.27	up
Propionylcarnitine	C10H19NO4	217.13	2.50	3.38	1.76	0.03	0.75	1.24	up
N-(4-butyl-2-methylphenyl)-N'-[4-(4-methylpiperazino)phenyl]urea	C23H32N4O	380.25	8.77	3.03	1.60	0.03	0.78	1.20	up
Oxaceprol	C7H11NO4	173.07	1.36	1.82	0.86	0.03	0.76	1.31	up
3-hydroxy-3-methylpentanedioic acid	C6H10O5	179.08	4.73	6.98	2.80	0.03	0.72	1.25	up
(2E)-4-Hydroxy-3,7-dimethyl-2,6-octadien-1-yl beta-D-glucopyranoside	C16H28O7	349.21	4.94	2.31	1.21	0.03	0.72	1.18	up
D-(+)-Maltose	C12H22O11	364.10	1.33	0.46	-1.11	0.03	0.72	1.19	down
RLH	C18H32N8O4	212.13	4.64	2.75	1.46	0.03	0.72	1.22	up
Proline-hydroxyproline	C10H16N2O4	228.11	5.12	1.77	0.82	0.03	0.76	1.25	up
PC (16:0/17:1)	C41H80NO8P	745.56	8.84	1.78	0.84	0.03	0.74	1.34	up
Gly-Tyr-Ala	C14H19N3O5	309.13	4.61	2.22	1.15	0.03	0.74	1.28	up
Estrone	C18H22O2	287.19	7.93	0.42	-1.25	0.03	0.72	1.32	down
Acetyl-L-carnitine	C9H17NO4	203.12	1.79	1.77	0.83	0.03	0.74	1.19	up
2-(2,4-dihydroxyphenyl)-3,5,7-trihydroxy-4H-chromen-4-one	C15H10O7	302.04	5.47	0.63	-0.66	0.03	0.69	1.37	down
ACar 20:0	C27H54NO4	455.40	8.37	2.79	1.48	0.03	0.72	1.40	up
1,4-dihydroxyheptadec-16-en-2-yl acetate	C19H36O4	328.26	8.89	15.57	3.96	0.03	0.67	1.19	up
8-Aminooctanoic acid	C8H17NO2	159.13	1.38	2.10	1.07	0.03	0.70	1.16	up
D-Galactosamine	C6H14ClNO5	215.06	1.31	0.65	-0.62	0.03	0.74	1.36	down

R-1 Methanandamide phosphate	C23H40NO5P	441.27	4.94	0.39	-1.35	0.03	0.71	1.14	down
LPE 15:1	C20H40NO7P	437.25	7.93	0.40	-1.33	0.03	0.83	1.34	down
13,14-dihydro-15-keto-PGD2	C20H32O5	352.22	6.76	0.53	-0.92	0.03	0.72	1.14	down
Oleoyl ethylamide	C20H39NO	309.30	10.47	1.76	0.81	0.03	0.69	1.22	up
5-Hydroxymethylcytosine	C5H7N3O2	141.05	1.81	2.54	1.34	0.03	0.69	1.17	up
ACar 7:0	C14H28NO4	273.19	5.51	3.17	1.67	0.03	0.70	1.15	up
D-Ala-D-Ala	C6H12N2O3	160.09	1.38	1.89	0.92	0.03	0.69	1.20	up
3-(propan-2-yl)-octahydropyrrolo[1,2-a]pyrazine-1,4-dione	C10H16N2O2	196.12	5.25	1.99	0.99	0.04	0.73	1.22	up
1-Methylnicotinamide	C7H8N2O	154.07	1.30	1.71	0.77	0.04	0.76	1.22	up
2-Linoleoyl glycerol	C21H38O4	336.26	10.15	2.20	1.14	0.04	0.72	1.14	up
Tauro-alpha-Muricholic acid sodium salt	C26H44NNaO7S	537.27	6.78	2.79	1.48	0.04	0.59	1.31	up
Oleoyl ethanolamide	C20H39NO2	325.30	9.58	1.87	0.90	0.04	0.72	1.16	up
Oxohongdenafil	C25H32N6O4	480.26	5.40	0.44	-1.19	0.04	0.74	1.28	down
Glycolithocholic acid	C26H43NO4	433.32	9.10	8.07	3.01	0.04	0.68	1.12	up
Nobiletin	C21H22O8	402.13	6.43	0.51	-0.97	0.04	0.70	1.12	down
PC (20:4e/2:0)	C30H54NO7P	571.36	9.30	1.70	0.77	0.04	0.75	1.22	up
ACar 13:0	C20H40NO4	357.29	6.44	0.61	-0.71	0.04	0.68	1.14	down
β-Nicotinamide mononucleotide	C11H15N2O8P	334.06	1.38	0.42	-1.27	0.05	0.76	1.23	down
LPE 16:1	C21H42NO7P	451.27	8.54	0.40	-1.33	0.05	0.67	1.25	down
3-amino-2-phenyl-2H-pyrazolo[4,3-c]pyridine-4,6-diol	C12H10N4O2	242.08	5.79	1.52	0.61	0.05	0.73	1.23	up
6??-Hydroxytestosterone	C19H28O3	304.20	7.83	2.32	1.22	0.05	0.73	1.09	up
Heptadecanoic Acid	C17H34O2	292.24	8.08	0.64	-0.65	0.05	0.74	1.13	down
