# Supplementary material for: Identification of Canine Pyometra-Associated Metabolites Using Untargeted Metabolomics
Source: Int J Mol Sci. 2022 Nov 16;23(22):14161. doi: 10.3390/ijms232214161 (PMC9697130; doi:10.3390/ijms232214161)
Supplement: Supplementary file 1 [file ijms-23-14161-s001.zip › Table S2.pdf]

Table S2. Differential metabolites identified in negative ion mode of endometrium of diseased and healthy dogs.

Name	Formula	Molecular Weight	RT [min]	FC	log2FC	P-value	ROC	VIP	Up/ Down
Levodopa	C9H11NO4	197.07	2.45	0.29	-1.80	0.00	0.94	2.23	down
D-Ribulose 5-phosphate	C5H11O8P	230.02	1.51	0.46	-1.11	0.00	0.93	2.03	down
Phe-Pro	C14H18N2O3	262.13	5.17	4.55	2.19	0.00	0.92	2.08	up
D-Glucose 6-phosphate	C6H13O9P	260.03	1.51	0.33	-1.58	0.00	0.90	1.84	down
(R)-3-Hydroxy myristic acid	C14H28O3	244.20	8.11	1.71	0.77	0.00	0.88	1.79	up
Lauric acid ethyl ester	C14H28O2	228.21	9.31	2.28	1.19	0.00	0.89	1.76	up
Prolylleucine	C11H20N2O3	228.15	5.10	4.24	2.08	0.00	0.87	1.70	up
Vitamin B2	C17H20N4O6	376.14	9.31	1.76	0.82	0.00	0.86	1.69	up
Hypoxanthine	C5H4N4O	136.04	2.00	0.63	-0.66	0.00	0.87	1.64	down
D-(-)-Fructose	C6H12O6	180.06	1.24	0.54	-0.89	0.00	0.86	1.63	down
L-Threonic acid	C4H8O5	136.04	1.34	0.52	-0.95	0.00	0.84	1.71	down
D-Sedoheptulose 7-phosphate	C7H15O10P	290.04	1.51	0.53	-0.92	0.00	0.84	1.67	down
2-deoxyglucose-6-phosphate	C6H13O8P	244.03	1.52	0.55	-0.87	0.00	0.84	1.65	down
N-Acetyl-L-methionine	C7H13NO3S	191.06	5.26	0.62	-0.68	0.00	0.82	1.94	down
(5-L-Glutamyl)-L-Amino Acid	C8H14N2O5	218.09	1.38	2.64	1.40	0.00	0.84	1.57	up
Prostaglandin H2	C20H32O5	334.21	7.61	2.10	1.07	0.00	0.84	1.56	up
Kaempferitrin	C27H30O14	624.17	9.08	0.42	-1.23	0.00	0.82	1.55	down
N-Acetylaspartic acid	C6H9NO5	175.05	1.94	0.34	-1.57	0.00	0.81	1.65	down
LPC 24:4	C32H58NO7P	645.40	9.98	3.05	1.61	0.00	0.80	1.55	up
2-Hydroxycaproic acid	C6H12O3	132.08	5.64	3.10	1.63	0.00	0.81	1.51	up
Cholic acid	C24H40O5	408.29	7.15	2.88	1.53	0.00	0.83	1.49	up
LPC 22:4	C30H54NO7P	617.37	9.28	2.22	1.15	0.00	0.82	1.49	up

trans-Cinnamic acid	C9H8O2	148.05	5.65	8.84	3.14	0.00	0.79	1.51	up
N-(5-Aminopentyl)acetamide	C7H16N2O	144.13	5.05	2.01	1.01	0.00	0.81	1.53	up
(2-anilino-4-methyl-1,3-thiazol-	C18H16N2O2S	324.10	4.94	5.67	2.50	0.00	0.74	1.51	up
Phosphocholine	C5H15ClNO4	219.04	1.43	0.57	-0.81	0.00	0.80	1.42	down
o-Toluic Acid	C8H8O2	136.05	5.17	5.93	2.57	0.00	0.79	1.42	up
DL-4-Hydroxyphenyllactic acid	C9H10O4	182.06	5.17	5.99	2.58	0.00	0.80	1.42	up
lipoamide	C8H15NOS2	205.06	5.24	0.40	-1.31	0.00	0.83	1.38	down
Azelaic acid	C9H16O4	188.11	5.79	1.75	0.81	0.00	0.80	1.43	up
Nicotinamide adenine	C21H28N7O17	743.08	1.87	0.66	-0.59	0.00	0.83	1.38	down
L-threo-3-Phenylserine	C9H11NO3	181.07	5.49	0.34	-1.55	0.00	0.78	1.43	down
9-HpOTrE	C18H30O4	310.21	7.61	2.79	1.48	0.00	0.83	1.35	up
4-[2-(2-oxo-1-	C9H17N3O3S	248.10	1.37	2.29	1.19	0.00	0.80	1.39	up
Ala-trp	C14H17N3O3	275.13	5.18	3.09	1.63	0.01	0.78	1.44	up
N-Acetyl-aspartic acid	C6H9NO5	175.05	1.47	0.44	-1.17	0.01	0.78	1.51	down
Glutaric Acid	C5H8O4	132.04	1.31	0.42	-1.24	0.01	0.78	1.44	down
(±)12(13)-DiHOME	C18H34O4	314.25	8.39	6.65	2.73	0.01	0.80	1.39	up
Glycocholic acid hydrate	C26H45NO7	483.32	6.49	2.41	1.27	0.01	0.79	1.39	up
2-Hydroxy-4-	C5H10O3S	150.04	5.22	11.53	3.53	0.01	0.78	1.40	up
Monobutyl phthalate	C12H14O4	222.09	6.21	2.00	1.00	0.01	0.81	1.49	up
Hexadecanedioic acid	C16H30O4	286.21	7.59	4.08	2.03	0.01	0.76	1.34	up
Mag (18:1)	C21H40O4	356.29	9.63	1.76	0.82	0.01	0.77	1.34	up
Dihydroxyacetone phosphate	C3H7O6P	170.00	1.51	0.45	-1.14	0.01	0.78	1.33	down
Propionyl-L-carnitine	C10H19NO4	217.13	5.87	2.38	1.25	0.01	0.79	1.31	up
Ouabain	C29H44O12	584.28	4.93	0.43	-1.22	0.01	0.79	1.31	down

1-Palmitoyl-Sn-Glycero-3-	C24H50NO7P	495.33	9.59	1.56	0.64	0.01	0.77	1.31	up
LPC 17:0	C25H52NO7P	555.35	9.59	1.65	0.72	0.01	0.76	1.32	up
Hydroxyglutaric acid	C5H8O5	148.04	1.45	0.37	-1.42	0.01	0.76	1.41	down
Cynaropicrin	C19H22O6	392.14	6.17	1.97	0.98	0.01	0.80	1.28	up
(3R)-8-hydroxy-3-(4-	C16H14O4	270.09	5.30	0.32	-1.63	0.01	0.78	1.33	down
Indole-3-lactic acid	C11H11NO3	205.07	5.58	3.63	1.86	0.01	0.73	1.35	up
Thymidine	C10H14N2O5	242.09	4.84	0.40	-1.31	0.01	0.77	1.31	down
Dodecanedioic acid	C12H22O4	230.15	6.40	1.97	0.98	0.01	0.80	1.36	up
O-Phosphorylethanolamine	C2H8NO4P	141.02	1.28	0.63	-0.66	0.01	0.75	1.34	down
FAHFA (22:4/3:0)	C25H40O4	404.29	10.76	4.00	2.00	0.01	0.76	1.31	up
Delta-Tridecalactone	C13H24O2	212.18	6.69	1.92	0.94	0.01	0.77	1.27	up
(±)19(20)-DiHDPA	C22H34O4	362.25	8.24	1.71	0.77	0.01	0.79	1.27	up
α-D-Glucose-1,6-bisphosphate	C6H14O12P2	340.00	2.29	0.39	-1.35	0.01	0.76	1.35	down
Uridine	C9H12N2O6	244.07	2.27	0.55	-0.85	0.01	0.76	1.27	down
H-Tyr(3-I)-OH	C9H10INO3	306.97	5.10	0.50	-0.99	0.01	0.76	1.29	down
DL-Citrulline	C6H13N3O3	175.10	1.55	12.03	3.59	0.01	0.73	1.26	up
15(R),19(R)-Hydroxy	C20H34O6	406.21	6.29	0.55	-0.86	0.01	0.74	1.37	down
Homovanillic acid	C9H10O4	182.06	5.69	4.96	2.31	0.01	0.76	1.39	up
8-Isoprostaglandin F1β	C20H36O5	356.26	8.35	3.54	1.82	0.01	0.79	1.24	up
OxPC (18:1-18:2+2O)	C44H82NO10	861.57	11.09	2.65	1.40	0.01	0.77	1.21	up
6-Phosphogluconic acid	C6H13O10P	276.02	1.56	0.54	-0.88	0.01	0.76	1.25	down
Arachidonic acid	C20H32O2	304.24	9.73	0.66	-0.60	0.01	0.75	1.28	down
Phenylpyruvic acid	C9H8O3	164.05	5.17	3.88	1.96	0.02	0.76	1.26	up
4-Methylvaleric Acid	C6H12O2	116.08	5.80	1.77	0.82	0.02	0.74	1.18	up

3-(methylsulfanyl)-5H-	C10H8N4S	216.05	5.23	2.50	1.32	0.02	0.74	1.26	up
Salicylic acid	C7H6O3	138.03	5.96	2.25	1.17	0.02	0.77	1.19	up
2-Methoxyestradiol	C19H26O3	302.18	5.73	2.19	1.13	0.02	0.74	1.35	up
LPC 18:0	C26H54NO7P	523.36	9.99	1.51	0.59	0.02	0.75	1.26	up
Traumatic acid	C12H20O4	228.14	6.25	1.60	0.68	0.02	0.72	1.31	up
Lysopc 17:0	C25H52NO7P	509.35	10.00	1.52	0.61	0.02	0.75	1.26	up
Thromboxane B2	C20H34O6	370.24	6.29	0.63	-0.67	0.02	0.74	1.27	down
16-Hydroxyhexadecanoic acid	C16H32O3	254.22	9.55	2.10	1.07	0.02	0.76	1.28	up
gamma-Glutamylmethionine	C10H18N2O5S	278.09	4.85	2.21	1.14	0.02	0.74	1.18	up
Mycophenolic acid	C17H20O6	320.12	5.64	5.22	2.38	0.02	0.71	1.19	up
4-oxo-4,5,6,7-	C9H8O4	180.04	5.47	4.85	2.28	0.02	0.71	1.27	up
2-Hydroxyestradiol	C18H24O3	288.17	5.56	2.66	1.41	0.02	0.69	1.18	up
Deoxyguanosine	C10H13N5O4	267.10	2.87	0.64	-0.65	0.03	0.76	1.13	down
4-Guanidinobutanoate	C5H11N3O2	145.09	1.76	26.95	4.75	0.03	0.60	1.15	up
OxPC (18:1-18:1+1O(1Cyc))	C44H82NO9P	845.58	11.66	3.57	1.84	0.03	0.74	1.18	up
N-Acetyl-L-tyrosine	C11H13NO4	223.08	5.18	1.52	0.61	0.03	0.72	1.23	up
Sepiapterin	C9H11N5O3	237.08	1.38	0.14	-2.81	0.03	0.84	1.64	down
Phosphoenolpyruvic acid	C3H5O6P	167.98	1.84	0.34	-1.57	0.03	0.75	1.15	down
10-Undecenoic acid	C11H20O2	184.15	6.25	1.74	0.80	0.03	0.72	1.08	up
19(R)-Hydroxy prostaglandin	C20H36O6	372.25	6.19	0.46	-1.13	0.03	0.71	1.41	down
4-Methylphenol	C7H8O	108.06	5.03	12.13	3.60	0.03	0.75	1.29	up
LPC 20:2	C28H54NO7P	593.37	9.44	1.54	0.62	0.03	0.74	1.35	up
D-Glucuronic acid	C6H10O7	194.04	5.22	2.84	1.50	0.03	0.66	1.14	up
Dl-3-Hydroxynorvaline	C5H11NO3	133.07	1.37	23.86	4.58	0.04	0.60	1.08	up

α,α-Trehalose	C12H22O11	342.12	1.33	0.58	-0.78	0.04	0.74	1.13	down
3-amino-1H-pyrazolo[4,3-	C6H6N4O2	166.05	10.99	1.95	0.96	0.04	0.77	1.23	up
OxPC (18:0-18:2+2O)	C44H84NO10	863.59	11.36	2.63	1.40	0.04	0.70	1.12	up
3-Phosphoglyceric acid	C3H7O7P	185.99	4.79	0.58	-0.79	0.04	0.72	1.02	down
Glycerol 3-phosphate	C3H9O6P	172.01	1.32	0.46	-1.12	0.05	0.80	1.14	down
O1-morpholinocarbonyl-3-	C13H14F3N3O	363.10	5.44	0.46	-1.13	0.05	0.68	1.02	down
Reserpine	C33H40N2O9	608.28	5.24	0.45	-1.16	0.05	0.68	1.02	down
Ursolic acid	C30H48O3	456.36	9.24	2.86	1.51	0.05	0.86	1.11	up
