# Supplementary material for: Identification of Canine Pyometra-Associated Metabolites Using Untargeted Metabolomics
Source: Int J Mol Sci. 2022 Nov 16;23(22):14161. doi: 10.3390/ijms232214161 (PMC9697130; doi:10.3390/ijms232214161)
Supplement: Supplementary file 1 [file ijms-23-14161-s001.zip › Table S3.pdf]

Table S3. The enriched pathways of differential endometrial metabolism in the positive ionization mode.

Map Title	P value	x	y	n	N	Enrich Direct	Meta IDs
Galactose metabolism	0.029	2	2	20	114	Over	D-Galactosamine; D-Fructose 6-phosphate
cAMP signaling pathway	0.079	2	3	20	114	Over	Acetylcholine; L-Adrenaline
Glycerophospholipid metabolism	0.141	2	4	20	114	Over	Acetylcholine; Phosphoethanolamine
Nicotinate and nicotinamide metabolism	0.141	2	4	20	114	Over	1-Methylnicotinamide; β-Nicotinamide mononucleotide
Starch and sucrose metabolism	0.175	1	1	20	114	Over	D-Fructose 6-phosphate
alpha-Linolenic acid metabolism	0.175	1	1	20	114	Over	Jasmonic acid
Sphingolipid metabolism	0.175	1	1	20	114	Over	Phosphoethanolamine
Riboflavin metabolism	0.175	1	1	20	114	Over	Riboflavin
AMPK signaling pathway	0.175	1	1	20	114	Over	D-Fructose 6-phosphate
Adrenergic signaling in cardiomyocytes	0.175	1	1	20	114	Over	L-Adrenaline
Synaptic vesicle cycle	0.175	1	1	20	114	Over	Acetylcholine
Regulation of actin cytoskeleton	0.175	1	1	20	114	Over	Acetylcholine
Insulin secretion	0.175	1	1	20	114	Over	Acetylcholine
Glucagon signaling pathway	0.175	1	1	20	114	Over	D-Fructose 6-phosphate
Insulin resistance	0.175	1	1	20	114	Over	D-Fructose 6-phosphate
Salivary secretion	0.175	1	1	20	114	Over	Acetylcholine
Gastric acid secretion	0.175	1	1	20	114	Over	Acetylcholine
Pancreatic secretion	0.175	1	1	20	114	Over	Acetylcholine
Nicotine addiction	0.175	1	1	20	114	Over	Acetylcholine
African trypanosomiasis	0.175	1	1	20	114	Over	L-Kynurenine
Neuroactive ligand-receptor interaction	0.192	3	9	20	114	Over	UDP; Acetylcholine; L-Adrenaline
Tryptophan metabolism	0.211	2	5	20	114	Over	Kynurenic acid; L-Kynurenine

Vitamin B6 metabolism	0.321	1	2	20	114	Over	Pyridoxine
Carbon metabolism	0.321	1	2	20	114	Over	D-Fructose 6-phosphate
Sphingolipid signaling pathway	0.321	1	2	20	114	Over	Phosphoethanolamine
Cholinergic synapse	0.321	1	2	20	114	Over	Acetylcholine
Taste transduction	0.321	1	2	20	114	Over	Acetylcholine
Prolactin signaling pathway	0.321	1	2	20	114	Over	Estrone
Renin secretion	0.321	1	2	20	114	Over	L-Adrenaline
Central carbon metabolism in cancer	0.321	1	2	20	114	Over	D-Fructose 6-phosphate
Alanine, aspartate and glutamate metabolism	0.443	1	3	20	114	Over	L-Argininosuccinate
Regulation of lipolysis in adipocytes	0.443	1	3	20	114	Over	L-Adrenaline
Arginine biosynthesis	0.543	1	4	20	114	Over	L-Argininosuccinate
Purine metabolism	0.543	1	4	20	114	Over	Deoxyadenosine
Metabolic pathways	0.603	16	83	20	114	Over	Kynurenic acid; Pyridoxine; UDP; 4-Guanidinobutyric acid; Riboflavin; Phosphoethanolamine; 1-Methylnicotinamide; D-Ala-D-Ala; L-Adrenaline; D-Fructose 6-phosphate; L-Kynurenine; β-Nicotinamide mononucleotide; Estrone; L-Argininosuccinate; Jasmonic acid; Deoxyadenosine
Lysine degradation	1	1	5	20	114	Over	N6,N6,N6-Trimethyl-L-lysine
Histidine metabolism	1	1	5	20	114	Over	1-Methylhistidine
Tyrosine metabolism	1	1	5	20	114	Over	L-Adrenaline
Ovarian steroidogenesis	1	1	5	20	114	Over	Estrone

Map Title: Enriched KEGG pathway name; P value: P value of enrichment analysis; X: Number of differential metabolites associated with this pathway; y: Number of background (all) metabolites associated with this pathway; n: Number of differential metabolites annotated by KEGG; N: Number of background (all) metabolites of KEGG annotation; Enrich Direct: Enrichment direction, Over represents enrichment, i.e. $x/n > y/N$; Meta IDs: List of enriched metabolites.
