# Supplementary material for: Identification of Canine Pyometra-Associated Metabolites Using Untargeted Metabolomics
Source: Int J Mol Sci. 2022 Nov 16;23(22):14161. doi: 10.3390/ijms232214161 (PMC9697130; doi:10.3390/ijms232214161)
Supplement: Supplementary file 1 [file ijms-23-14161-s001.zip › Table S4.pdf]

Table S4. The enriched pathways of differential endometrial metabolism in the negative ionization mode.

MapTitle	P value	x	y	n	N	Enrich Direct	MetaIDs
Glycerophospholipid metabolism	0.065	3	4	31	111	Over	Phosphocholine; O-Phosphorylethanolamine; Dihydroxyacetone phosphate
Serotonergic synapse	0.065	3	4	31	111	Over	Arachidonic acid; Thromboxane B2; Prostaglandin H2
Glycolysis / Gluconeogenesis	0.076	2	2	31	111	Over	Phosphoenolpyruvic acid; Dihydroxyacetone phosphate
Starch and sucrose metabolism	0.076	2	2	31	111	Over	α,α-Trehalose; D-Glucose 6-phosphate
Platelet activation	0.076	2	2	31	111	Over	Arachidonic acid; Prostaglandin H2
Retrograde endocannabinoid signaling	0.076	2	2	31	111	Over	Arachidonic acid; Prostaglandin H2
Dopaminergic synapse	0.076	2	2	31	111	Over	Homovanillic acid; Levodopa
Prolactin signaling pathway	0.076	2	2	31	111	Over	Levodopa; D-Glucose 6-phosphate
Thyroid hormone synthesis	0.076	2	2	31	111	Over	Nicotinamide adenine dinucleotide phosphate; D-Glucose 6-phosphate
Arachidonic acid metabolism	0.132	3	5	31	111	Over	Arachidonic acid; Thromboxane B2; Prostaglandin H2
Metabolic pathways	0.17	26	81	31	111	Over	Arachidonic acid; 4-Guanidinobutanoate; Cholic acid; 4-Methylphenol; trans-Cinnamic acid; Salicylic acid; o-Toluic Acid; Phenylpyruvic acid; Deoxyguanosine; Uridine; Phosphocholine; Homovanillic acid; Nicotinamide adenine dinucleotide phosphate; α,α-Trehalose; Thromboxane B2; Phosphoenolpyruvic acid; Levodopa; D-Ribulose 5-phosphate; D-Sedoheptulose 7-phosphate; Hypoxanthine; Vitamin B2; D-

							Glucose 6-phosphate; Prostaglandin H2; Thymidine; O-Phosphorylethanolamine; Dihydroxyacetone phosphate
Pentose and glucuronate interconversions	0.188	2	3	31	111	Over	D-Ribulose 5-phosphate; Dihydroxyacetone phosphate
Phenylalanine, tyrosine and tryptophan biosynthesis	0.188	2	3	31	111	Over	Phenylpyruvic acid; Phosphoenolpyruvic acid
Inositol phosphate metabolism	0.188	2	3	31	111	Over	D-Glucose 6-phosphate; Dihydroxyacetone phosphate
Riboflavin metabolism	0.188	2	3	31	111	Over	D-Ribulose 5-phosphate; Vitamin B2
Ovarian steroidogenesis	0.188	2	3	31	111	Over	Arachidonic acid; 2-Hydroxyestradiol
Oxytocin signaling pathway	0.188	2	3	31	111	Over	Arachidonic acid; Prostaglandin H2
Carbon metabolism	0.216	4	8	31	111	Over	Phosphoenolpyruvic acid; D-Ribulose 5-phosphate; D-Sedoheptulose 7-phosphate; Dihydroxyacetone phosphate
Bile secretion	0.263	4	9	31	111	Over	Cholic acid; Salicylic acid; Ouabain; Thromboxane B2
Fructose and mannose metabolism	0.279	1	1	31	111	Over	Dihydroxyacetone phosphate
Ubiquinone and other terpenoid-quinone biosynthesis	0.279	1	1	31	111	Over	trans-Cinnamic acid
Phosphonate and phosphinate metabolism	0.279	1	1	31	111	Over	Phosphoenolpyruvic acid
Glycerolipid metabolism	0.279	1	1	31	111	Over	Dihydroxyacetone phosphate
Linoleic acid metabolism	0.279	1	1	31	111	Over	Arachidonic acid
Sphingolipid metabolism	0.279	1	1	31	111	Over	O-Phosphorylethanolamine
Propanoate metabolism	0.279	1	1	31	111	Over	Dihydroxyacetone phosphate
Folate biosynthesis	0.279	1	1	31	111	Over	Sepiapterin

Sphingolipid signaling pathway	0.279	1	1	31	111	Over	O-Phosphorylethanolamine
Necroptosis	0.279	1	1	31	111	Over	Arachidonic acid
Vascular smooth muscle contraction	0.279	1	1	31	111	Over	Arachidonic acid
Fc gamma R-mediated phagocytosis	0.279	1	1	31	111	Over	Arachidonic acid
Long-term depression	0.279	1	1	31	111	Over	Arachidonic acid
Inflammatory mediator regulation of TRP channels	0.279	1	1	31	111	Over	Arachidonic acid
Insulin secretion	0.279	1	1	31	111	Over	D-Glucose 6-phosphate
GnRH signaling pathway	0.279	1	1	31	111	Over	Arachidonic acid
Carbohydrate digestion and absorption	0.279	1	1	31	111	Over	D-Glucose 6-phosphate
Parkinson's disease	0.279	1	1	31	111	Over	Levodopa
Cocaine addiction	0.279	1	1	31	111	Over	Levodopa
Amphetamine addiction	0.279	1	1	31	111	Over	Levodopa
Alcoholism	0.279	1	1	31	111	Over	Levodopa
Pentose phosphate pathway	0.311	2	4	31	111	Over	D-Ribulose 5-phosphate; D-Sedoheptulose 7-phosphate
Tyrosine metabolism	0.311	2	4	31	111	Over	Homovanillic acid; Levodopa
Phenylalanine metabolism	0.346	3	6	31	111	Over	trans-Cinnamic acid; Salicylic acid; Phenylpyruvic acid
Primary bile acid biosynthesis	0.482	1	2	31	111	Over	Cholic acid
Lysine degradation	0.482	1	2	31	111	Over	Glutaric Acid
Neomycin, kanamycin and gentamicin biosynthesis	0.482	1	2	31	111	Over	D-Glucose 6-phosphate

alpha-Linolenic acid metabolism	0.482	1	2	31	111	Over	Traumatic acid
Ferroptosis	0.482	1	2	31	111	Over	Arachidonic acid
Fc epsilon RI signaling pathway	0.482	1	2	31	111	Over	Arachidonic acid
Regulation of lipolysis in adipocytes	0.482	1	2	31	111	Over	Arachidonic acid
Choline metabolism in cancer	0.482	1	2	31	111	Over	Phosphocholine
Biosynthesis of amino acids	0.511	5	13	31	111	Over	Phenylpyruvic acid; Phosphoenolpyruvic acid; D-Ribulose 5-phosphate; D-Sedoheptulose 7-phosphate; Dihydroxyacetone phosphate
Aldosterone synthesis and secretion	0.617	2	5	31	111	Over	Arachidonic acid; Nicotinamide adenine dinucleotide phosphate
Nicotinate and nicotinamide metabolism	0.671	2	6	31	111	Over	Nicotinamide adenine dinucleotide phosphate; Dihydroxyacetone phosphate
Galactose metabolism	1	1	3	31	111	Over	Dihydroxyacetone phosphate
Fatty acid degradation	1	1	3	31	111	Over	Glutaric Acid
Arginine and proline metabolism	1	1	3	31	111	Over	4-Guanidinobutanoate
Pyruvate metabolism	1	1	3	31	111	Over	Phosphoenolpyruvic acid
Vitamin B6 metabolism	1	1	3	31	111	Over	D-Ribulose 5-phosphate
Insulin resistance	1	1	3	31	111	Over	D-Glucose 6-phosphate
Central carbon metabolism in cancer	1	1	3	31	111	Over	D-Glucose 6-phosphate
Pyrimidine metabolism	1	2	7	31	111	Over	Uridine; Thymidine

Map Title: Enriched KEGG pathway name; P value: P value of enrichment analysis; X: Number of differential metabolites associated with this pathway; y: Number of background (all) metabolites associated with this pathway; n: Number of differential metabolites annotated by KEGG; N: Number of background (all) metabolites of KEGG annotation; Enrich Direct: Enrichment direction, Over represents enrichment, i.e. x/n ＞ y/N; Meta IDs: List of enriched metabolites.
