# Supplementary material for: Identification of Canine Pyometra-Associated Metabolites Using Untargeted Metabolomics
Source: Int J Mol Sci. 2022 Nov 16;23(22):14161. doi: 10.3390/ijms232214161 (PMC9697130; doi:10.3390/ijms232214161)
Supplement: Supplementary file 1 [file ijms-23-14161-s001.zip › Table S5.pdf]

Table S5 The clinical information of diseased dogs with pyometra and healthy dogs.

Health status of dogs	Endometrial tissue	Breed	Lineage	Somatotype	Age (years)
Cannine pyometra	PP001	Samoyed	Pure	Medium	10
Cannine pyometra	PP002	Poodle	Pure	Small	16
Cannine pyometra	PP003	Poodle	Pure	Small	2
Cannine pyometra	PP004	Teddydog	Pure	Small	12
Cannine pyometra	PP005	Cocker	Pure	Small	11
Cannine pyometra	PP006	Bobbie	Pure	Small	10
Cannine pyometra	PP007	Border Collie	Pure	Medium	4
Cannine pyometra	PP008	Italian Greyhound	Pure	Small	9
Cannine pyometra	PP009	Teddydog	Pure	Small	8
Cannine pyometra	PP010	Pomeranian	Pure	Small	12
Cannine pyometra	PP011	Teddydog	Pure	Small	7
Cannine pyometra	PP012	Old English Sheepdog	Pure	Large	9
Cannine pyometra	PP013	Teddydog	Pure	Small	10
Cannine pyometra	PP014	Teddydog	Pure	Small	8
Cannine pyometra	PP015	Golden retriever	Pure	Large	12
Heathy	HD001	Pomeranian	Pure	Small	11
Heathy	HD002	Schnauzer	Pure	Small	3
Heathy	HD003	Poodle	Pure	Small	8
Heathy	HD004	Pomeranian	Pure	Small	8
Heathy	HD005	Teddydog	Pure	Small	7
Heathy	HD006	Samoyed	Pure	Medium	6
Heathy	HD007	Teddydog	Pure	Small	8
Heathy	HD008	Teddydog	Pure	Small	8
Heathy	HD009	Teddydog	Pure	Small	12
Heathy	HD010	Teddydog	Pure	Small	4
Heathy	HD011	Border Collie	Pure	Medium	9
Heathy	HD012	Pomeranian	Pure	Small	11
Heathy	HD013	Border Collie	Pure	Medium	9
Heathy	HD014	Teddydog	Pure	Small	10
Heathy	HD015	Teddydog	Pure	Small	9
